# Two Cases of Massively Prolapsed Patent Vitellointestinal Duct

**DOI:** 10.21699/ajcr.v8i2.534

**Published:** 2017-03-18

**Authors:** Sudhir Singh, Digamber Chaubey, JD Rawat, Gurmeet Singh

**Affiliations:** Department of Pediatric Surgery, King George's Medical University, Lucknow, India

**Keywords:** Patent vitellointestinal duct, Prolapsed intussusception, Intestinal obstruction

## Abstract

Patent vitellointestinal duct (PVID) is a benign congrnital anomaly ususally presenting with fecal discharge from the umbilicus. In this report, we describe two cases of PVID presented with massive bowel prolapse through the PVID and signs of intestinal obstruction. Surgery revealed prolapse of the ileal intussusceptum through the PVID. Both of the babies were sucssesfully managed with surgery.

## CASE REPORT

Case 1: A full term, first born, male baby presented to us at day 20 of life with some feculent discharge from his umbilicus for ten days. The umbilicus revealing a red prolapsing mass and there was a small quantity of faecal discharge. Examination showed a soft, distended abdomen with a small tubular mucosal lined structure at the umbilicus (Fig.1A). Parents refuted surgical management at that time. At two months of age, the baby presented with bilious vomiting, dehydration, and a bowel mass protruding from the umbilicus (Fig.1B). After stabilization, he underwent trans-umbilical exploration. Operative finding were prolapsed ileal intussusceptum through PVID with small segment of compromised ileum. Patient managed with reduction of the prolapsed bowel and primary anastomosis with resection of PVID along with segment of compromised small bowel.(Fig.1C,1D). The patient's immediate postoperative period and follow up of up to six months were uneventfull. Histopathological examination of the excised specimen suggestive of small bowel tissue that was consistent with PVID without any evidence of ectopic mucosa.

**Figure:**
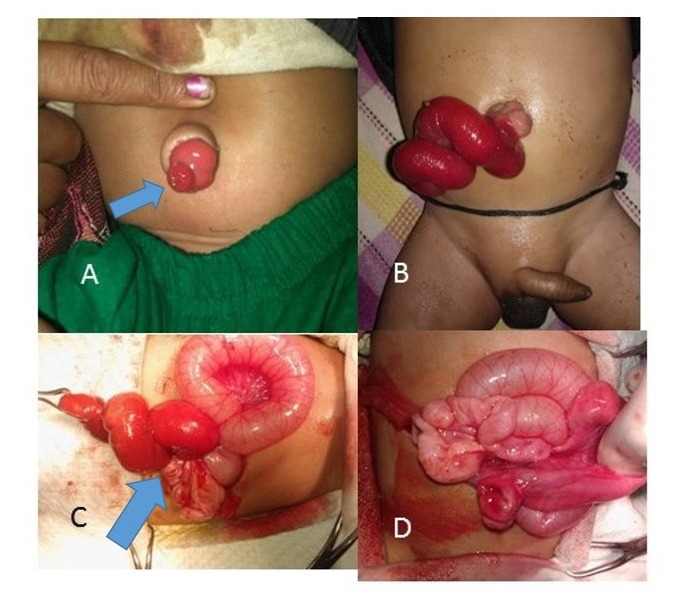
Figure 1: photograph of case no 1, A showing uncomplicated PVID, B showing prolapsed distal bowel through PVID, C intraoperative photographs, D after reduction of distal bowel intussusception.

Case 2: A 1.5-month old male baby presented in our emergency with bowel mass protruding from umbilicus, abdominal distention, and vomiting since last six hours (Fig.2A). Baby was first born full term with normal antenatal scan. Parents noticed that baby has small red protruding mass at umbilicus with small faecal discharge since day 12 of life. Baby was malnourished with weight 2.2 kg. Baby had no associated anomalies. Baby underwent trans-umbilical exploration with reduction of the prolapsed ileal intussusceptum through PVID and primary anastomosis with resection of PVID along with segment of compromised small bowel were done. (Fig.2B,2C). Postoperative course was uneventful.

**Figure:**
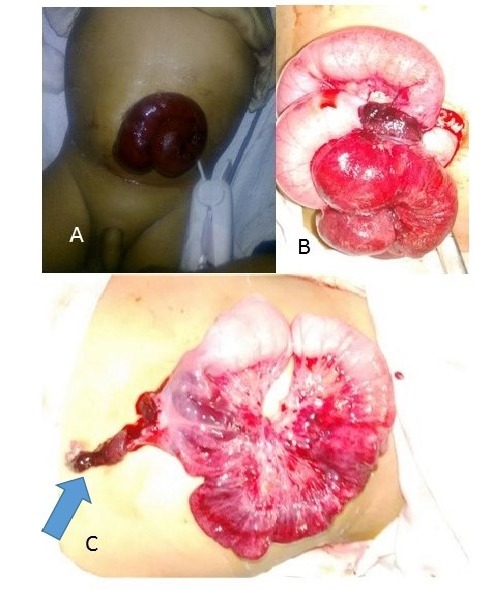
Figure 2: photograph of case no 2, A showing prolapsed distal bowel through PVID, B intraoperative photographs, C after reduction of distal bowel intussusception.

## DISCUSSION

Vitellointestinal duct usually obliterates by the end of the seventh week of gestation. Persistence of this gives rise to a spectrum of congenital anomalies - sinus, cyst, fistula, band, Meckels diverticulum. The incidence of PVID is 0.0053%.[1] PVID seen eight times more common in males and mostly present at neonatal periods as our both cases are male.[2] The presentation is with fecal discharge from umbilicus through a mucosal lined tubulr structure. It is a benigh condition with very straightforward surgical treatment (Transumbilical mobilisation and resection of PVID with ileal anastomosis). Delay in treatment can cause bleeding and mucosal prolapse through PVID.[3,4] Massive prolapse can occur leading to intestinal obstruction. Massive prolapse through narrowed umbilical ring also obliterates bowel lumen leading to mechanical bowel obstruction. In severe cases of massive mucosal prolapse, bowel ischemia may ensue thus necessitating bowel resection and anastomsois.[3] Only few of the previous reported cases prolapsed intussuscepted distal small bowel via patent vitellointestinal duct were found. [1-5] Factors responsible for prolapsed of bowel may be wide weak umbilical defect, condition that cause increase intraabdominal pressure along with partial bowel obstruction at PVID sites. In view of these serious consequences, PVID should be treated promptly. To conclude, the presentation of PVID may be varied form minor skin excoriations to lethal bowel obstruction and strangulations. Cases needing early surgical correction should be identified and corrected promptly.

## Footnotes

**Source of Support:** Nil

**Conflict of Interest:** None declared

